# Predictive Model for Managing the Clinical Risk of Emergency Department Patients: A Systematic Review

**DOI:** 10.3390/jcm14207245

**Published:** 2025-10-14

**Authors:** Maria João Baptista Rente, Liliana Andreia Neves da Mota, Ana Lúcia da Silva João

**Affiliations:** 1 Self-Care and Patient-Centered Care (Patient Care), Comprehensive Health Research Center, Universidade de Évora, Palácio dos Colegiais 2, 7004-516 Évora, Portugal; ana.joao@essaude.ipsantarem.pt; 2 Serviço de Urgência Médico-Cirúrgico, Departamento de Urgência e Emergência, Unidade Local de Saúde do Litoral Alentejano (ULSLA), Monte do Gilbardinho, 7540-230 Santiago do Cacém, Portugal; 3 Conselho Técnico-Científico, Escola Superior de Saúde Norte da Cruz Vermelha Portuguesa, Rua da Cruz Vermelha Cidacos—Apartado 1002, 3720-126 Oliveira de Azeméis, Portugal; liliana.mota@essnortecvp.pt; 4 LT3—Ciência de Dados, de Decisão & Tecnologias de Informação, Tech4edusim—Technologies for Education and Simulation in Healthcare, CINTESIS: Centro de Investigação em Tecnologias e Serviços de Saúde, Escola Superior de Enfermagem do Porto, Rua Dr. António Bernardino de Almeida, 830, 844, 856, 4200-072 Porto, Portugal; 5 Enfermagem, Escola Superior de Saúde de Santarém, Instituto Politécnico de Santarém, Quinta do Mergulhão, Srª da Guia, 2005-075 Santarém, Portugal

**Keywords:** predictive learning models, risk management, emergency service, hospital

## Abstract

**Background/Objective**: The growing volume and complexity of cases presented to emergency departments underline the urgent need for effective clinical-risk-management strategies. Increasing demands for quality and safety in healthcare highlight the importance of predictive tools in supporting timely and informed clinical decision-making. This study aims to evaluate the performance and usefulness of predictive models for managing the clinical risk of people who visit the emergency department. **Methods**: A systematic review was conducted, including primary observational studies involving people aged 18 and over, who were not pregnant, and who had visited the emergency department; the intervention was clinical-risk management in emergency departments; the comparison was of early warning scores; and the outcomes were predictive models. Searches were performed on 10 November 2024 across eight electronic databases without date restrictions, and studies published in English, Portuguese, and Spanish were included in this study. Risk of bias was assessed using the Checklist for Critical Appraisal and Data Extraction for Systematic Reviews of Prediction Modeling Studies as well as the Prediction Model Risk-of-Bias Assessment Tool. The results were synthesized narratively and are summarized in a table. **Results**: Four studies were included, each including between 4388 and 448,972 participants. The predictive models identified included the Older Persons' Emergency Risk Assessment score; a new situation awareness model; machine learning and deep learning models; and the Vital-Sign Scoring system. The main outcomes evaluated were in-hospital mortality and clinical deterioration. **Conclusions**: Despite the limited number of studies, our results indicate that predictive models have potential for managing the clinical risk of emergency department patients, with the risk-of-bias study indicating low concern. We conclude that integrating predictive models with artificial intelligence can improve clinical decision-making and patient safety.

## 1. Introduction

The purpose of emergency departments (EDs) is to provide care to people at clinical risk of experiencing complex disease processes and/or organ failure [1,2,3,4,5,6].

Emergency departments require transdisciplinary approaches [1,2,3,4,7,8] that combine diverse fields of expertise [4,9] to anticipate clinical risk and standardize assessment processes [2,3,5,6,10,11].

Risk stratification [1,2,6,10,12,13,14,15], supported by predictive models, can improve the anticipation of patient needs and enhance their safety and quality of care [2,6,9,10,12,13,14,16,17,18].

These approaches are aligned with current evidence highlighting the importance of proactive risk management in acute care settings [2,3,5,6,10,11].

A predictive model can support emergency professionals in systematizing decisions and organizing care in transdisciplinary teams [1,2,3,4,5,6,7,8,11,19,20,21,22,23,24,25].

Predictive models, typically grounded in algorithmic frameworks derived from machine learning or statistical methodologies, are designed to estimate risk and thereby inform decision-making processes in EDs [21,22,24,26,27,28].

Machine learning approaches, which include supervised and unsupervised techniques such as logistic regression, decision trees, ensemble learning, and neural networks, enable the identification of complex patterns in large clinical datasets. These models have shown increasing promise in predicting outcomes such as hospital admission, intensive care needs, and mortality, complementing or outperforming traditional scoring systems [29]. Their integration into emergency care may facilitate earlier recognition of deterioration and more targeted interventions.

The objective of this review is to evaluate the performance and usefulness of predictive models for managing the clinical risk of people who visit the ED.

## 2. Materials and Methods

This systematic review was conducted and reported according to the Preferred Reporting Items for Systematic Reviews and Meta-Analyses (PRISMA) [30,31] (Appendix A). We performed a systematic review on the use of predictive models for managing the clinical risk of ED patients. 

**Registration and protocol:** This protocol was registered in PROSPERO under the name “Predictive model for managing the clinical risk of ED patients: A systematic review” and registration number CRD42024556926. No protocol was prepared, and no amendments have been made to the information provided at registration.

### 2.1. Eligibility Criteria

We aimed to identify all predictive models developed until 10 November 2024 for managing the clinical risk of ED patients. The articles needed to fulfill the following population, intervention, comparison, and outcome criteria to be considered for inclusion (Table 1).

### 2.2. Information Sources

The following databases were searched from inception until 10 November 2024: CINAHL^®^ Plus, the Health Technology Assessment Database, MedicLatina, MED-LINE^®^, PubMed, Scopus, the Cochrane Plus Collection, and Web of Science.

Information sources were restricted to peer-reviewed articles to ensure methodological rigor and reliability of the extracted data, and the gray literature and preprints were excluded.

The selection of electronic databases was informed by guidance from the Cochrane Handbook for Systematic Reviews of Interventions (Chapter 4: Searching for and selecting studies) [33]. MedicLatina was included due to its coverage of peer-reviewed scientific and medical journals from established Latin American and Spanish-language publishers, thereby enabling comprehensive retrieval of the relevant literature published in Spanish.

### 2.3. Search Strategy

Among the entry terms, we used the following keywords according to Medical Subject Headings: risk assessment (health risk assessment, risk analysis); risk management; risk adjustment (case-mix adjustment); risk factors; early warning score; emergency service; and hospital (emergency departments). The initial search strategy was developed in PubMed and subsequently adapted to meet the specific syntax and indexing requirements of each included database. The full search strategies for all databases are provided in Appendix B.

### 2.4. Selection Process

Duplicate articles were removed using Rayyan. Two authors (M.R. and L.M.) independently screened titles and abstracts, and conflicting results were discussed in consensus meetings. After screening the titles and abstracts, the full text of each article was assessed for eligibility by the same authors to decide whether or not they should be included in the systematic review.

### 2.5. Data Collection Process

The method used to collect data from the included articles followed the guidelines of the Cochrane Handbook for Systematic Reviews of Interventions [33]. One author (M.R.) collected data from each article, and a random check was conducted by another author (L.M.). This check showed no discrepancies.

### 2.6. Data Items

The following data were extracted from every included article: study details (authors, publication year, language, country where the study was carried out, study aim/research question, design, recruitment source, inclusion and exclusion criteria, type of allocation, stratification, and sample size); characteristics of participants (age, gender, ethnicity, and multimorbidities); intervention details (intervention content, intervention setting, delivery of intervention, and number of participants assessed at follow-up); comparison/control characteristics (type of control program/intervention); and outcomes (primary and secondary outcomes, where between-group differences, total scores/means, and standard deviations in each group were extracted from the study results; methods of outcome measurement, including blinding procedures; and time of outcome measurements).

### 2.7. Risk-of-Bias Assessment in Included Studies

The methods used to assess risk of bias in the included studies were assessed with the Critical Appraisal and Data Extraction for Systematic Reviews of Prediction Modeling Studies (CHARMS) checklist and Prediction Model Risk-of-Bias Assessment Tool (PROBAST) [34]. Two review authors (M.R. and L.M.) independently assessed the risk of bias in the included studies, and disagreements were resolved by consensus.

### 2.8. Effect Measures

The effect measures that we evaluated for the outcomes of predictive models for clinical-risk management were discrimination and calibration.

### 2.9. Synthesis Methods

We visually displayed the results of individual studies and syntheses in a table, which groups the characteristics of the included studies, including the authors, year, sample, objectives, assessment tools, interventions, results, and conclusions.

The method used to synthesize the results and our rationale for choosing this method are reported in the text.

Owing to the anticipated methodological and clinical heterogeneity, a meta-analysis was not performed. Instead, a narrative synthesis was undertaken to address heterogeneity across model types, with particular emphasis on identifying and interpreting inconsistencies in the findings—such as divergent effect directions or substantial variations in effect magnitudes—across the included studies.

### 2.10. Reporting Bias Assessment

To assess potential reporting biases, selective outcome reporting within studies was evaluated by comparing the reported results with those outlined in the study protocols, where available. Although a formal meta-analysis was not conducted, the potential for publication bias was considered by documenting the presence or absence of non-significant findings and assessing whether studies were prospectively registered (e.g., in clinical trial registries). Any discrepancies between registered protocols and final publications will be critically appraised and discussed.

### 2.11. Certainty Assessment

The methods used in the included studies to assess confidence in the body of evidence for an outcome were herein assessed using the GRADE (Grading of Recommendations Assessment, Development, and Evaluation) approach [35,36,37], which evaluates the quality of evidence based on factors such as risk of bias, consistency, directness, precision, and publication bias.

## 3. Results

### 3.1. Study Selection

**Figure 1:**
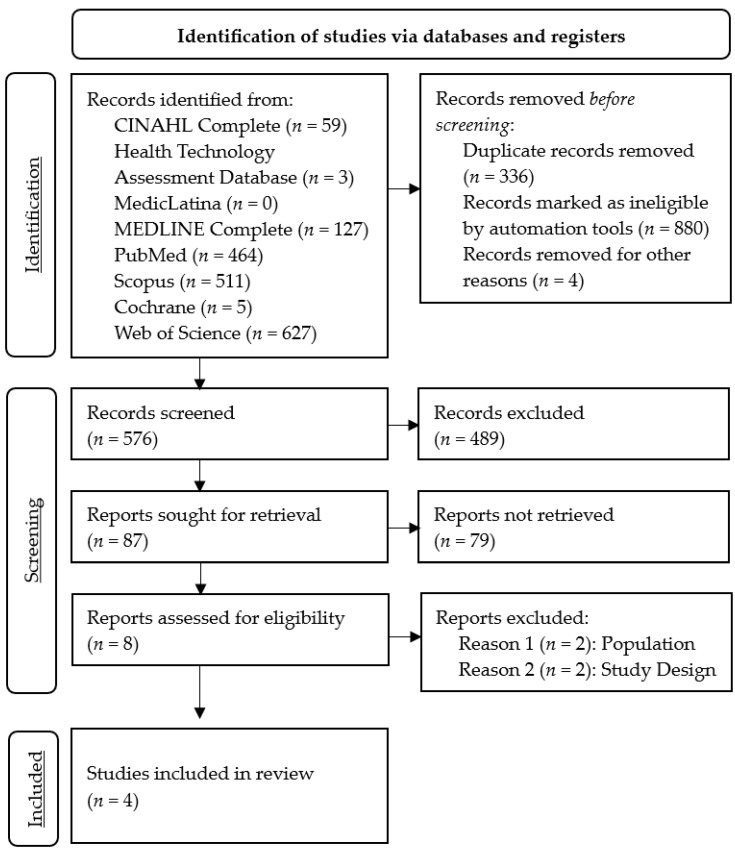
PRISMA 2020 flow diagram for systematic review on the use of predictive models for managing the clinical risk of ED patients.

The search process identified 1796 records, of which 576 remained after the removal of records before screening. The exclusion of studies based on titles and abstracts resulted in the retention of eight full-text articles eligible for assessment. Of these, four articles were excluded because the studies did not address the population criteria (*n =* 2) or the study design criteria (*n =* 2). Finally, we included four articles in this systematic review, with full details of the study selection summarized in Figure 1.

### 3.2. Study Characteristics

The study characteristics are summarized in Table 2. The four studies utilized different models: the Older Persons' Emergency Risk Assessment (OPERA) score, which is a risk prediction model developed and validated by the authors to predict in-hospital mortality and other outcomes in older adults admitted to the ED [38]; the new situation awareness (SA) model, which was introduced in an intervention group and consisted of the existing regional early warning score (EWS) system plus five additional subjective parameters (skin observations, dyspnea reported by the patient, pain, clinical intuition or concern, and patients' or relatives' concerns) [39]; benchmarking ED prediction models, which refer to various machine learning and deep learning models (including logistic regression, random forest, gradient boosting, multilayer perception, Med2Vec, and long short-term memory) that were developed and evaluated for predicting three different ED outcomes [40]; and the Vital-Sign Scoring (VSS) system, which is based on the presence of seven potential vital-sign abnormalities that were used to predict hospital mortality [41].

Three of the included studies had a prospective design, and all four studies prospectively assessed the performance of the models.

### 3.3. Risk of Bias in Studies

The results of the risk-of-bias assessment for each included study are presented in Table 3 and Supplementary Materials, for which we used the CHARMS checklist and PROBAST [34].

In [38], selection bias may have occurred due to the exclusion of patients without NEWS2 or CFS data, as well as patients with a short length of stay, and there was potential information bias due to missing data, which the authors addressed through multiple imputations. In general, the study design and methods aimed to help to reduce the risk of bias, but there are still potential sources of bias that could affect the validity of the results.

In [39], seasonal variation in the case mix and imbalance in the baseline characteristics between the intervention and control groups may have influenced the results. In addition, there was a lack of fidelity monitoring to ensure that the intervention was implemented as planned.

In [40], selection bias due to the single-center nature of the dataset may have limited the generalizability of the results, and measurement bias and residual confounding may have occurred due to the lack of certain potentially important risk factors in the dataset. In addition, bias could have been introduced by the simple imputation method used to handle missing data, which can hide the underlying data structure.

Based on the information provided in [41], the primary sources of bias in this study are likely selection bias due to the exclusion of outpatient cases, and information bias due to the need to extract data from patient records in some cases. However, the authors recognize these limitations and provide evidence that they are unlikely to have had a significant impact on the results. Overall, the risk of bias in this study appears to be moderate.

### 3.4. Results of Individual Studies

The OPERA risk score was derived and validated using data of 8974 and 8391 patients, respectively. The OPERA model included the NEWS2, CFS, acute kidney injury, age, sex, and the Malnutrition Universal Screening Tool (MUST). The OPERA model demonstrated superior performance for predicting in-hospital mortality, with an area under the curve (AUC) of 0.79, compared to NEWS2 (AUC = 0.65) and CFS (AUC = 0.76). The OPERA risk groups were able to predict prolonged hospital stay, with the highest-risk group having an odds ratio of 9.7 for staying more than 30 days. The OPERA model maintained good performance even when excluding the variable requiring a laboratory result (creatinine for acute kidney injury) [38].

The new SA model reduced the odds of CD by 21% compared to the control group, but there was no significant impact on mortality, ICU admissions, or readmissions [39].

The gradient boosting machine learning model achieved high performance in predicting critical outcomes (Area Under the Receiver Operating Characteristic—AUROC: 0.880) and hospitalization (AUROC: 0.819), but lower performance in predicting 72-h ED reattendance, and deep learning models did not outperform the gradient boosting model. Traditional clinical scoring systems had poor discriminatory performance, but the interpretable AutoScore model achieved better performance in predicting critical outcomes (AUROC 0.846) and hospitalization (AUROC 0.793) using a small number of variables [40].

**Figure 2:**
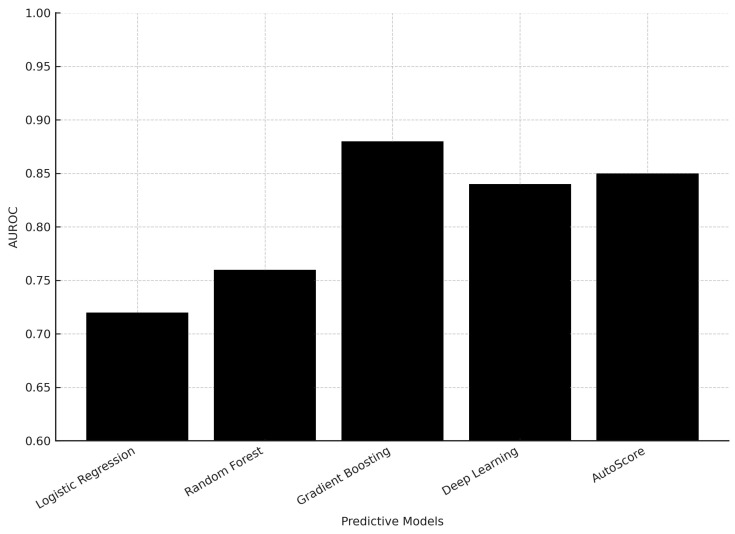
Comparison of AUROC performance of predictive models for ED risk management.

Figure 2 illustrates the comparison of AUROC performance across predictive models, highlighting the superior performance of gradient boosting.

Most individual vital-sign abnormalities, except seizures and abnormal respiratory rate, were independent predictors of hospital mortality. Higher initial and maximum VSS values were significantly associated with increased hospital mortality. VSS values had a higher predictive power for hospital mortality when collected in the first 15 min after ED admission, compared to the maximum score over the entire stay [41].

### 3.5. Results of Syntheses

The OPERA model performed even better at predicting in-hospital mortality when excluding patients already receiving care or palliative care, with an AUC of 0.80 in the validation cohort [38].

Machine learning models, particularly gradient boosting, outperformed other methods in predicting hospitalization and critical outcomes, but struggled with predicting 72-h ED reattendance. While traditional clinical scoring systems performed poorly, the interpretable AutoScore model achieved reasonably good performance on the critical outcome and hospitalization prediction tasks [40].

### 3.6. Reporting Biases

None of the studies explicitly discuss any communication biases.

### 3.7. Certainty of Evidence

Based on the study design, methods, and statistical analysis, the certainty of evidence in [39] is moderate. The quasi-experimental controlled pre- and post-intervention design, large sample size, and appropriate statistical analysis provide a reasonable level of confidence in the findings, but the inherent limitations of a non-randomized study design prevent this study from achieving higher certainty.

Based on the details provided in [41], the certainty of evidence appears to be high. This study was a large, prospective cohort study with a comprehensive set of relevant patient data, conducted ethically and with appropriate oversight. The methodological details suggest a high-quality study with a high degree of certainty in its findings.

The methods used in the included studies to assess confidence in the body of evidence for an outcome were herein assessed using the GRADE (Grading of Recommendations Assessment, Development, and Evaluation) approach [35,36,37] (Table 4).

We are moderately confident that an estimate of the effect (or association) is correct (i.e., the certainty of the evidence from the included studies is moderate). Our confidence in the effect estimate is limited by the small number of included studies, and the true effect may be substantially different from the effect estimate.

## 4. Discussion

### 4.1. Current Research Status

The first study derived and validated the OPERA score, which can help clinicians stratify older patients by risk of mortality and prolonged hospital stay. The model demonstrated good discrimination and calibration, with a small over-prediction of mortality risk, which was addressed. Excluding patients without a documented frailty score limited the sample size, as this assessment may not have been conducted for patients in relatively good health. OPERA performed better for short-term mortality compared to longer-term outcomes, likely due to its focus on acute-illness severity. OPERA provided useful odds ratios for extended hospital stay, which could support discharge planning and resource allocation [38,42].

The new SA model reduced the odds of CD compared to the existing EWS system, but did not impact secondary outcomes like mortality, ICU admission, or readmissions. The SA model’s wider approach to increasing situational awareness and identifying early signs of deterioration was supported by these findings. The lack of impact on mortality is consistent with previous studies on EWS systems. There was a trend towards reduced ICU admissions in the intervention group, but this was likely due to differences in case mix. A higher EWS at ED entry was associated with increased risk of CD, as seen in other studies [28,39,43,44].

Machine learning models demonstrated higher predictive accuracy than traditional scoring systems, but complex deep learning models did not outperform simpler models on the relatively low-dimensional ED data. While machine learning models had higher predictive accuracy, their black-box nature makes them less suitable for clinical decision-making in emergency care, where explainability is important. Traditional scoring systems had lower predictive accuracy, but the interpretable AutoScore system achieved higher accuracy while maintaining the advantages of transparent, point-based scoring systems [19,40].

VSS, which measures the presence, onset, or worsening of vital-sign instability, is highly predictive of hospital mortality. Both the initial VSS value and its change during the ED stay is relevant, with patients who exhibit an increase in VSS having higher mortality. The individual components of VSS, such as impaired consciousness, hypotension, hypoxemia, and abnormal heart rate, were the strongest predictors of mortality. Its lack of independent predictive value for seizures and respiratory rate may be due to their co-occurrence with other VSS components. These results suggest that using VSS for the rapid identification and treatment of at-risk patients in EDs has the potential to improve outcomes for critically ill patients [41,45].

### 4.2. Trends

Gradient boosting methods demonstrated superior AUROC values (0.819–0.880) compared to logistic regression and random forest, while deep learning models did not outperform simpler methods. This suggests that moderately complex models may provide the best balance between predictive accuracy and interpretability in emergency settings. Clinical integration requires models that are not only accurate but also interpretable at the bedside. Tools such as AutoScore provide transparent, point-based frameworks that maintain predictive accuracy while allowing clinicians to understand and trust the decision-making process—an essential factor for adoption in high-pressure emergency settings.

Table 5 provides a schematic overview summarizing the strengths and limitations of traditional scoring systems, machine learning, and deep learning approaches.

### 4.3. Limitations

The limitations of the evidence obtained in this study are related to the scarcity of existing studies on predictive models for managing the clinical risk of ED patients. Furthermore, the number of included studies was small, which might be because we only included studies in English, Portuguese, and Spanish. Importantly, the small number of studies included significantly limits the generalizability of our findings.

In practice, predictive models represent objective methods to identify clinical risk or deterioration, supporting timely decision-making and interventions to improve patient outcomes.

### 4.4. Future Prospects

Future research should focus on integrating explainable machine learning models into electronic health record systems to ensure real-time applicability in clinical workflows. Transparent frameworks, such as AutoScore, may balance predictive performance with interpretability, thereby increasing acceptance among healthcare professionals. Moreover, multicenter external validation studies are essential to confirm generalizability across diverse emergency department populations.

This study contributes to transforming the care model in health services, with re-percussions for health policies that will make it possible to reorganize EDs.

## 5. Conclusions

This systematic review highlights the potential of predictive models in managing clinical risk in EDs. Despite the limited number of studies included, our findings demonstrate that models such as OPERA, machine learning models, the situation awareness model, and the VSS system represent valuable solutions for predicting in-hospital mortality and clinical deterioration. Their implementation may support faster and more informed clinical decisions, as well as optimize resource allocation in high-pressure care settings. However, the strength of our conclusions is limited by the inclusion of only four studies, underscoring the need for more multicenter validation research.

The integration of predictive models with artificial intelligence tools represents an opportunity to significantly enhance patient safety and the quality of care they receive. However, the widespread adoption of such methods requires robust external validation, adaptation to the realities of different emergency services, and special attention to the clinical interpretability of models—an essential condition for their acceptance by healthcare professionals.

Therefore, future research should focus on the development and validation of transparent, efficient predictive models that are integrated into clinical information systems, contributing to a more proactive, safe, and person-centered care paradigm.

## Figures and Tables

**Table 1:** Population, intervention, comparison, outcomes, and study (PICOS) design framework [32], as well as year and language criteria.

PICOS Framework	Elements	Description
Population	People aged 18 and over, who were not pregnant, and who had visited the ED	-
Intervention	Clinical risk management in EDs	Clinical-risk management refers to the process of minimizing liability exposure in healthcare settings by focusing on safety, security, and quality of patient care.
Comparison	Early warning score	Early warning scores are simple tools that help detect clinical deterioration to improve patient safety in hospitals.
Outcome	Predictive model	Predictive models are machine learning models which are trained to analyze historical data to find patterns and trends, allowing them to predict future outcomes. Due to the exploratory nature and inclusive approach of this review, predictive models will be eligible regardless of whether they have undergone internal or external validation. However, the validation status of each model will be recorded and discussed.
Study Design	We will include primary observational studies, while preprints and retracted articles will be excluded	-
Year and Language	Articles in Portuguese, Spanish, and English will be included, with no limit on publication time	Only studies published in English, Portuguese, or Spanish will be included. This may limit the comprehensiveness of the review but aligns with the language capabilities of the review team.

**Table 2:** Characteristics of the studies included for systematic review of the use of predictive models for managing the clinical risk of ED patients (*n =* 4).

Study	Clinical Decision-Making in Older Adults Following Emergency Admission to Hospital. Derivation and Validation of a Risk Stratification Score: OPERA [38]	A New Situation Awareness Model Decreases Clinical Deterioration in the Emergency Departments—A Controlled Intervention Study [39]	Benchmarking Emergency Department Prediction Models with Machine Learning and Public Electronic Health Records [40]	Risk Assessment in the First Fifteen Minutes: A Prospective Cohort Study of a Simple Physiological Scoring System in the Emergency Department [41]
*Study Details*				
Authors	Arjan et al [38]	Tygesen et al [39]	Xie et al [40]	Merz et al [41]
Publication year	2021	2021	2022	2011
Language	English	English	English	English
Country where the study was carried out	United Kingdom	Denmark	Not mentioned (this paper does not explicitly state the country where this study was carried out)	Switzerland
Study aim/research question	To derive and validate a risk score for acutely unwell older adults, which may enhance risk stratification and support clinical decision-making. How can we develop a risk stratification score for older adults admitted to the hospital that can enhance risk prediction and support clinical decision-making?	To investigate the effect on clinical deterioration (CD) of a new SA model including EWS, combined with skin observation; clinical concern and patients’ and relatives’ concerns; pain and dyspnea reported by patients; and risk assessment by the medical team. Does a new SA model consisting of objective and subjective parameters, compared to a conventional EWS system, reduce clinical deterioration in adult ED patients?	To standardize data preprocessing and establish a comprehensive ED benchmark dataset alongside comparable risk prediction models for three ED-based outcomes. What is the best way to develop a standardized benchmark dataset and prediction tasks for EDs based on a large electronic database of public health records, to facilitate reproducibility, model comparison, and progress in applying machine learning to emergency care?	To assess the incidence of measurable vital-sign abnormalities at admission to the ED and the potential impact of these factors on treatment delay and outcomes in a large group of unselected patients needing hospital admission. What is the incidence of measurable vital-sign abnormalities at admission to the ED, and can a scoring system based on these vital signs predict treatment delay and patient outcomes?
Study design	Prospective, multi-site observational study with a derivation cohort and an external validation cohort. It used regression modeling to derive and validate a risk prediction score for in-hospital mortality in older adults admitted through the ED.	Controlled, multi-site, prospective, pre and post-intervention study.	Not mentioned (the study design is not explicitly mentioned in this study).	Prospective, observational cohort study of 4388 consecutive adult patients admitted to the ED of a tertiary referral hospital over 6 months.
Recruitment source	ED admissions at two hospitals on the south coast of England that are part of the same hospital organization.	- Referral by a general practitioner (GP) or out-of-hours GP service; - Arrival by ambulance after an emergency call; - Self-referral (for a small number of participants).	Not mentioned (this paper does not mention the source of recruitment by the authors or re-searchers).	All adult patients admitted to the ED of Bern University Hospital between 11 June 2007, and 11 January 2008.
Inclusion and exclusion criteria	Inclusion criteria: patients aged 65 years or older who were admitted through the EDs of the two hospitals. Exclusion criteria: patients who did not have a recorded National Early Warning Score 2 (NEWS2) or clinical frailty scale (CFS) score, or whose hospital stay was less than 1 day.	Inclusion criteria: - Patients ≥ 18 years old; - Patients with medical or surgical complaints; - Patients admitted to the ED short stay unit; - Only the first admission during the study period was included. Exclusion criteria: - Patients discharged home within 4 h of arrival; - Patients referred to inpatient wards within 4 h of arrival.	Inclusion criteria: - Patients 18 years of age or older; - Patients assigned to a primary emergency triage class; Exclusion criteria: - Patients under 18 years old; - Patients not assigned to a primary emergency triage class. The dataset was also split into a 20% test set and an 80% training set, with the test set fixed so that future researchers can use it for model comparisons.	Inclusion criteria: all patients admitted to the ED of the Bern University Hospital between 11 June 2007, and 11 January 2008. Exclusion criteria: patients treated on an outpatient basis and patients with missing data on vital-sign abnormalities.
Type of allocation	Not mentioned (this paper does not mention the type of allocation used in this study).	Controlled pre- and post-intervention study, with two EDs assigned to the intervention group and two EDs assigned to the control group.	Not mentioned (this paper does not mention the type of allocation for this study).	Observational, as it was a prospective cohort study with no allocation of participants to different treatment groups.
Stratification	The development and validation of the OPERA risk score was designed to stratify older adult patients admitted through the ED into different risk groups for in-hospital mortality and pro-longed hospital stay.	Patients were stratified based on their EWS at admission to determine whether the intervention had a different effect depending on the patients’ initial condition.	Not mentioned (this paper does not mention any stratification methods or analysis).	The VSS system used two main scores—the initial VSS value in the first 15 minutes after ED admission, and the maximum VSS value (VSS max) during the entire ED stay. Patients were stratified based on these scores, which were found to be strongly predictive of hospital mortality, with the initial VSS value being the most predictive.
Sample size	The total sample size was 17,365 participants, with 8974 in the derivation cohort and 8391 in the validation cohort.	The total sample size was 34,556 patients, after excluding 7281 patients with a length of stay less than 4 h.	The total sample size included 448,972 ED visits by 216,877 unique patients. The test set consisted of 88,287 ED episodes (20% of the total), and the training set consisted of the remaining 80% of ED episodes.	4388 patients.
*Characteristics of Participants*				
Age	Adults aged 65 and older.	Adult patients aged 18 and above. Pre (years): - Intervention A—63; - Intervention B—63; - Control C—66; - Control D—64. Post (years): - Intervention A—66; - Intervention B—64; - Control C—70; - Control D—66.	Adult patients aged 18 and older.	Adult patients aged 61.
Gender	Male (%): 4088 (45.6%); female: 3856 (46.0%).	Pre (Female): - Intervention A—1897; - Intervention B—2377; - Control C—1188; - Control D—3034. Post (Female): - Intervention A—2266; - Intervention B—2736; - Control C—1182; - Control D—3064.	Female: - Overall: 239,794 (54.3%). - Outcomes: - Discharge: 133,874 (57.6%); - Hospitalized: 105,920 (50.7%); . Critical outcomes: 12,168 (46.5%); - 72-h ED reattendance: 7068 (46.2%). Male: - Overall: 201,643 (45.7%). - Outcomes: - Discharge: 98,587 (42.4%); Hospitalized: 103,056 (49.3%); - Critical outcomes: 14,006 (53.5%); - 72-h ED reattendance: 8231 (53.8%).	Not mentioned (this paper does not mention gender).
Ethnicity	Not mentioned (this paper does not mention the ethnicity of the study participants).	Not mentioned (this paper does not mention the ethnicity of the study participants).	Not mentioned (this paper does not mention the ethnicity of the study participants).	Not mentioned (this paper does not mention the ethnicity of the study participants).
Multimorbidities	The multimorbidities included in the OPERA risk score model were congestive cardiac failure, diabetes, liver disease, and chronic kidney disease.	Not mentioned (this paper does not mention the multimorbidities of the participants).	The multimorbidities of patients were measured using the Charlson Comorbidity Index (CCI) and Elixhauser Comorbidity Index (ECI) based on the International Classification of Diseases (ICD) diagnosis codes in the Medical Information Mart for Intensive Care IV (MIMIC-IV) ED database. The authors also used a neural network-based embedding approach like Med2Vec to incorporate the comorbidity information into their predictive models.	Not mentioned (this paper does not mention anything about the multimorbidities of the participants).
*Intervention Details*				
Intervention content	Not mentioned (this paper does not describe any specific intervention content).	The intervention content was a new SA model that included: (1) the regional EWS system; (2) additional parameters like skin observations, patient-reported symptoms, clinical intuition, and patient/relative concerns; (3) a process where nurses checked for deterioration and called physicians if needed; and 4) twice-daily risk assessments by the medical team to discuss at-risk patients.	Not mentioned (this paper does not describe any specific intervention content).	Not mentioned (this paper does not describe any specific intervention content).
Intervention setting	The interventions took place at two non-specialist hospital EDs on the south coast of England, within the same NHS Trust.	The intervention took place at four regional EDs in the Central Den-mark Region, with two EDs as-signed to the intervention group and two to the control group.	Not mentioned (this paper does not mention an "intervention setting" as it is a methodological study that describes the creation of a benchmark dataset and the evaluation of various prediction models on that dataset).	The intervention took place at the ED of a 960-bed tertiary referral hospital.
Delivery of intervention	Not mentioned (this paper does not mention the delivery of a specific intervention).	The intervention consisted of implementing a new SA model in intervention-group EDs. The model included the existing regional EWS system plus five additional parameters: skin observations, dyspnea reported by the patient, new or increasing pain, clinical intuition or concern, and patients' or relatives' concerns. Nurses were trained to consider deterioration to have occurred if either the EWS was triggered or one of the five additional parameters was present, and to call a physician if deterioration was observed. At-risk patients were highlighted on electronic dashboards and discussed during twice-daily risk assessments by the medical team.	Not mentioned (this paper does not mention the delivery of a specific intervention).	Not mentioned (this paper does not mention the delivery of a specific intervention).
Number of participants assessed at follow-up points	Not mentioned (this paper does not mention the number of participants assessed at follow-up).	21,839 participants were assessed at follow-up.	Not mentioned (this paper does not mention the number of participants assessed at follow-up, as it does not appear to involve any longitudinal follow-up of participants).	Not mentioned (this paper does not mention the number of participants assessed at follow-up, as it appears to have focused on the initial assessment and outcomes during patients’ hospital stay rather than follow-up data collection).
Comparison/Control Characteristics				
Type of control program/interventions	Not mentioned (this paper does not mention any type of control program or intervention).	A new SA model was introduced in the intervention group, which added five additional parameters (skin observations, dyspnea, pain, clinical intuition/concern, and patient/relative concern) to the existing regional EWS system. The intervention group received training on the new SA model, while the control group continued using the existing regional EWS system.	Not mentioned (this paper does not mention any type of control program or intervention).	Not mentioned (this paper does not mention any type of control program or intervention).
Outcomes				
Primary outcomes	In-hospital mortality.	- CD, defined as a change in vital signs that required increased observation or assessment by a physician, i.e., an increase in regional EWS from 0 or 1 to ≥2 or an in-crease from ≥2; - A composite outcome of CD combined with death or Intensive Care Unit (ICU) admission directly from the ED.	- Hospitalization—inpatient admission immediately following an ED visit; - Critical outcome—inpatient mortality or transfer to the ICU within 12 hours; - ED reattendance—return visit to the ED within 72 h of previous discharge.	Hospital mortality.
Secondary outcomes	48-hour mortality, 7-day mortality, hospital stay >30 days, and readmission after <30 days of discharge.	- Proportion of 30-day readmissions; - Proportion of 7-day mortality; - Proportion of 30-day mortality; - Proportion of ICU admissions.	Not mentioned (this paper does not mention any secondary outcomes).	Combined endpoint of ICU admission or death in the ED.

**Table 3:** Risk-of-bias and applicability assessments of the development studies.

Author, Year	Risk of Bias				Applicability			Overall	
	Participants	Predictors	Outcome	Analysis	Participants	Predictors	Outcome	Risk of Bias	Applicability
Arjan et al., 2021 [38]	+	+	+	+	+	+	+	+	+
Tygesen et al., 2021 [39]	+	+	+	+	+	+	+	+	+
Xie et al., 2022 [40]	+	+	+	+	+	+	+	+	+
Merz et al., 2011 [41]	+	+	+	+	+	+	+	+	+

+ Low concern.

**Table 4:** Grade indicating quality of evidence.

Author, Year	Grade
Arjan et al., 2021 [38]	++
Tygesen et al., 2021 [39]	+++
Xie et al., 2022 [40]	++
Merz et al., 2011 [41]	++++

++++ high; +++ moderate; ++ low.

**Table 5:** Overview of predictive models in ED risk management.

Predictive Models	Description
Traditional Scoring Systems	• Simple, interpretable • Limited predictive power
Machine Learning Models	• Higher accuracy • Risk of black-box effect
Deep Learning Models	• Handle large datasets • Less interpretable in clinical practice
Hybrid/Interpretable Models (for example, AutoScore)	• Balance accuracy and transparency • More suitable for bedside adoption

## Data Availability

All data are provided via tables in the text, and via text and tables in the appendices. The included articles are available via the electronic databases utilized in this study.

## Supplementary Materials

The following supporting information can be downloaded at: https://www.mdpi.com/article/10.3390/jcm14207245/s1, CHARMS and PROBAST templates.

## Abbreviations

The following abbreviations are used in this manuscript:

AUC	Area Under the Curve
AUROC	Area Under the Receiver Operating Characteristic
CCI	Charlson Comorbidity Index
CD	Clinical Deterioration
CFS	Clinical Frailty Scale
CHARMS	Critical Appraisal and Data Extraction for Systematic Reviews of Prediction Modeling Studies
ECI	Elixhauser Comorbidity Index
ED	Emergency Department
EWS	Early Warning Score
GP	General Practitioner
GRADE	Grading of Recommendations Assessment, Development, and Evaluation
ICD	International Classification of Diseases
ICU	Intensive Care Unit
Med2Vec	Multi-layer Representation Learning Tool for Medical Concepts
MIMIC-IV	Medical Information Mart for Intensive Care IV
MUST	Malnutrition Universal Screening Tool
NEWS2	National Early Warning Score 2
NHS	National Health Services
OPERA	Older Persons' Emergency Risk Assessment
PICOS	Population, Intervention, Comparison, Outcomes, and Study
PRISMA	Preferred Reporting Items for Systematic Reviews and Meta-Analyses
PROBAST	Prediction Model Risk-of-Bias Assessment Tool
PROSPERO	International Prospective Register of Systematic Reviews
SA	Situation Awareness
VSS	Vital-Sign Scoring

## Appendix A

**Table A1:** PRISMA 2020 checklist.

Section and Topic	Item #	Checklist Item	Location Where Item Is Reported
**TITLE**			
Title	1	Identify the report as a systematic review.	Page 1 Lines 2 and 3
**ABSTRACT**			
Abstract	2	See the PRISMA 2020 for Abstracts checklist.	Page 1 Lines 18 to 41
**INTRODUCTION**			
Rationale	3	Describe the rationale for the review in the context of existing knowledge.	Page 2 Lines 46 to 67
Objectives	4	Provide an explicit statement of the objective(s) or question(s) the review addresses.	Page 2 Lines 68 and 69
**METHODS**			
Eligibility criteria	5	Specify the inclusion and exclusion criteria for the review and how studies were grouped for the syntheses.	Page 3 Lines 79 to 84 Table 1
Information sources	6	Specify all databases, registers, websites, organizations, reference lists and other sources searched or consulted to identify studies. Specify the date when each source was last searched or consulted.	Page 4 Lines 86 to 96
Search strategy	7	Present the full search strategies for all databases, registers and websites, including any filters and limits used.	Page 4 Lines 98 to 104
Selection process	8	Specify the methods used to decide whether a study met the inclusion criteria of the review, including how many reviewers screened each record and each report retrieved, whether they worked independently, and if applicable, details of automation tools used in the process.	Page 4 Lines 106 to 110
Data collection process	9	Specify the methods used to collect data from reports, including how many reviewers collected data from each report, whether they worked independently, any processes for obtaining or confirming data from study investigators, and if applicable, details of automation tools used in the process.	Page 4 Lines 112 to 115
Data items	10a	List and define all outcomes for which data were sought. Specify whether all results that were compatible with each outcome domain in each study were sought (e.g. for all measures, time points, analyses), and if not, the methods used to decide which results to collect.	Page 4 Lines 117 to 126
	10b	List and define all other variables for which data were sought (e.g. participant and intervention characteristics, funding sources). Describe any assumptions made about any missing or unclear information.	Page 4 Lines 117 to 126
Study risk of bias assessment	11	Specify the methods used to assess risk of bias in the included studies, including details of the tool(s) used, how many reviewers assessed each study and whether they worked independently, and if applicable, details of automation tools used in the process.	Pages 4 to 5 Lines 128 to 132
Effect measures	12	Specify for each outcome the effect measure(s) (e.g. risk ratio, mean difference) used in the synthesis or presentation of results.	Page 5 Lines 134 to 135
Synthesis methods	13a	Describe the processes used to decide which studies were eligible for each synthesis (e.g. tabulating the study intervention characteristics and comparing against the planned groups for each synthesis (item #5)).	Page 5 Lines 137 to 146
	13b	Describe any methods required to prepare the data for presentation or synthesis, such as handling of missing summary statistics, or data conversions.	Page 5 Lines 137 to 146
	13c	Describe any methods used to tabulate or visually display results of individual studies and syntheses.	Page 5 Lines 137 to 146
	13d	Describe any methods used to synthesize results and provide a rationale for the choice(s). If meta-analysis was performed, describe the model(s), method(s) to identify the presence and extent of statistical heterogeneity, and software package(s) used.	Page 5 Lines 137 to 146
	13e	Describe any methods used to explore possible causes of heterogeneity among study results (e.g. subgroup analysis, meta-regression).	Page 5 Lines 137 to 146
	13f	Describe any sensitivity analyses conducted to assess robustness of the synthesized results.	Page 5 Lines 137 to 146
Reporting bias assessment	14	Describe any methods used to assess risk of bias due to missing results in a synthesis (arising from reporting biases).	Page 5 Lines 148 to 154
Certainty assessment	15	Describe any methods used to assess certainty (or confidence) in the body of evidence for an outcome.	Page 5 Lines 156 to 160
**RESULTS**			
Study selection	16a	Describe the results of the search and selection process, from the number of records identified in the search to the number of studies included in the review, ideally using a flow diagram.	Page 5 and 6 Lines 163 to 171 Figure 1
	16b	Cite studies that might appear to meet the inclusion criteria, but which were excluded, and explain why they were excluded.	Page 5 and 6 Lines 163 to 171 Figure 1
Study characteristics	17	Cite each included study and present its characteristics.	Page 6 to 12 Lines 173 to 188 Table 2
Risk of bias in studies	18	Present assessments of risk of bias for each included study.	Page 13 Lines 191 to 213 Table 3
Results of individual studies	19	For all outcomes, present, for each study: (a) summary statistics for each group (where appropriate) and (b) an effect estimate and its precision (e.g. confidence/credible interval), ideally using structured tables or plots.	Pages 13 and 14 Lines 215 to 243 Figure 2
Results of syntheses	20a	For each synthesis, briefly summarize the characteristics and risk of bias among contributing studies.	Page 14 to 15 Lines 245 to 252
	20b	Present results of all statistical syntheses conducted. If meta-analysis was done, present for each the summary estimate and its precision (e.g. confidence/credible interval) and measures of statistical heterogeneity. If comparing groups, describe the direction of the effect.	Page 14 to 15 Lines 245 to 252
	20c	Present results of all investigations of possible causes of heterogeneity among study results.	Page 14 to 15 Lines 245 to 252
	20d	Present results of all sensitivity analyses conducted to assess the robustness of the synthesized results.	Page 14 to 15 Lines 245 to 252
Reporting biases	21	Present assessments of risk of bias due to missing results (arising from reporting biases) for each synthesis assessed.	Page 15 Line 254
Certainty of evidence	22	Present assessments of certainty (or confidence) in the body of evidence for each outcome assessed.	Page 15 Lines 256 to 273 Table 4
**DISCUSSION**			
Discussion	23a	Provide a general interpretation of the results in the context of other evidence.	Page 15 to 17 Lines 275 to 340 Table 5
	23b	Discuss any limitations of the evidence included in the review.	Page 15 to 17 Lines 275 to 340 Table 5
	23c	Discuss any limitations of the review processes used.	Page 15 to 17 Lines 275 to 340 Table 5
	23d	Discuss implications of the results for practice, policy, and future research.	Page 15 to 17 Lines 275 to 340 Table 5
**OTHER INFORMATION**			
Registration and protocol	24a	Provide registration information for the review, including register name and registration number, or state that the review was not registered.	Page 17 Lines 366 to 369
	24b	Indicate where the review protocol can be accessed, or state that a protocol was not prepared.	Page 17 Lines 366 to 369
	24c	Describe and explain any amendments to information provided at registration or in the protocol.	Page 17 Lines 366 to 369
Support	25	Describe sources of financial or non-financial support for the review, and the role of the funders or sponsors in the review.	Page 17 Lines 370 and 371
Competing interests	26	Declare any competing interests of review authors.	Page 18 Line 382
Availability of data, code and other materials	27	Report which of the following are publicly available and where they can be found: template data collection forms; data extracted from included studies; data used for all analyses; analytic code; any other materials used in the review.	Page 18 Lines 375 and 376

## Appendix B

### Appendix B.1. CINAHL^®^ Plus

(risk assessment OR Risk Assessments OR Assessment, Risk OR Health Risk Assessment OR Assessment, Health Risk OR Health Risk Assessments OR Risk Assessment, Health OR Risk Analysis OR Analysis, Risk OR Risk Analyses OR risk management OR Management, Risk OR Management, Risks OR Risks Management OR risk adjustment OR Adjustment, Risk OR Adjustments, Risk OR Risk Adjustments OR Case-Mix Adjustment OR Adjustment, Case-Mix OR Adjustments, Case-Mix OR Case Mix Adjustment OR Case-Mix Adjustments OR risk factors OR Factor, Risk OR Risk Factor) AND (early warning score OR Early Warning Scores OR Score, Early Warning OR Scores, Early Warning OR Warning Scores, Early) AND (emergency service, hospital OR Emergency Services, Hospital OR Hospital Emergency Services OR Services, Hospital Emergency OR Hospital Emergency Service OR Service, Hospital Emergency OR Emergency Hospital Service OR Emergency Hospital Services OR Hospital Service, Emergency OR Hospital Services, Emergency OR Service, Emergency Hospital OR Services, Emergency Hospital OR Hospital Service Emergency OR Emergencies, Hospital Service OR Emergency, Hospital Service OR Hospital Service Emergencies OR Service Emergencies, Hospital OR Service Emergency, Hospital OR Emergency Departments OR Department, Emergency OR Departments, Emergency OR Emergency Department)

### Appendix B.2. Health Technology Assessment Database

((risk assessment) OR (Risk Assessments) OR (Assessment, Risk) OR (Health Risk Assessment) OR (Assessment, Health Risk) OR (Health Risk Assessments) OR (Risk Assessment, Health) OR (Risk Analysis) OR (Analysis, Risk) OR (Risk Analyses) OR (risk management) OR (Management, Risk) OR (Management, Risks) OR (Risks Management) OR (risk adjustment) OR (Adjustment, Risk) OR (Adjustments, Risk) OR (Risk Adjustments) OR (Case-Mix Adjustment) OR (Adjustment, Case-Mix) OR (Adjustments, Case-Mix) OR (Case Mix Adjustment) OR (Case-Mix Adjustments) OR (risk factors) OR (Factor, Risk) OR (Risk Factor)) AND ((early warning score) OR (Early Warning Scores) OR (Score, Early Warning) OR (Scores, Early Warning) OR (Warning Scores, Early)) AND ((emergency service, hospital) OR (Emergency Services, Hospital) OR (Hospital Emergency Services) OR (Services, Hospital Emergency) OR (Hospital Emergency Service) OR (Service, Hospital Emergency) OR (Emergency Hospital Service) OR (Emergency Hospital Services) OR (Hospital Service, Emergency) OR (Hospital Services, Emergency) OR (Service, Emergency Hospital) OR (Services, Emergency Hospital) OR (Hospital Service Emergency) OR (Emergencies, Hospital Service) OR (Emergency, Hospital Service) OR (Hospital Service Emergencies) OR (Service Emergencies, Hospital) OR (Service Emergency, Hospital) OR (Emergency Departments) OR (Department, Emergency) OR (Departments, Emergency) OR (Emergency Department))

### Appendix B.3. MedicLatina

(risk assessment OR Risk Assessments OR Assessment, Risk OR Health Risk Assessment OR Assessment, Health Risk OR Health Risk Assessments OR Risk Assessment, Health OR Risk Analysis OR Analysis, Risk OR Risk Analyses OR risk management OR Management, Risk OR Management, Risks OR Risks Management OR risk adjustment OR Adjustment, Risk OR Adjustments, Risk OR Risk Adjustments OR Case-Mix Adjustment OR Adjustment, Case-Mix OR Adjustments, Case-Mix OR Case Mix Adjustment OR Case-Mix Adjustments OR risk factors OR Factor, Risk OR Risk Factor) AND (early warning score OR Early Warning Scores OR Score, Early Warning OR Scores, Early Warning OR Warning Scores, Early) AND (emergency service, hospital OR Emergency Services, Hospital OR Hospital Emergency Services OR Services, Hospital Emergency OR Hospital Emergency Service OR Service, Hospital Emergency OR Emergency Hospital Service OR Emergency Hospital Services OR Hospital Service, Emergency OR Hospital Services, Emergency OR Service, Emergency Hospital OR Services, Emergency Hospital OR Hospital Service Emergency OR Emergencies, Hospital Service OR Emergency, Hospital Service OR Hospital Service Emergencies OR Service Emergencies, Hospital OR Service Emergency, Hospital OR Emergency Departments OR Department, Emergency OR Departments, Emergency OR Emergency Department)

### Appendix B.4. MEDLINE^®^

(risk assessment OR Risk Assessments OR Assessment, Risk OR Health Risk Assessment OR Assessment, Health Risk OR Health Risk Assessments OR Risk Assessment, Health OR Risk Analysis OR Analysis, Risk OR Risk Analyses OR risk management OR Management, Risk OR Management, Risks OR Risks Management OR risk adjustment OR Adjustment, Risk OR Adjustments, Risk OR Risk Adjustments OR Case-Mix Adjustment OR Adjustment, Case-Mix OR Adjustments, Case-Mix OR Case Mix Adjustment OR Case-Mix Adjustments OR risk factors OR Factor, Risk OR Risk Factor) AND (early warning score OR Early Warning Scores OR Score, Early Warning OR Scores, Early Warning OR Warning Scores, Early) AND (emergency service, hospital OR Emergency Services, Hospital OR Hospital Emergency Services OR Services, Hospital Emergency OR Hospital Emergency Service OR Service, Hospital Emergency OR Emergency Hospital Service OR Emergency Hospital Services OR Hospital Service, Emergency OR Hospital Services, Emergency OR Service, Emergency Hospital OR Services, Emergency Hospital OR Hospital Service Emergency OR Emergencies, Hospital Service OR Emergency, Hospital Service OR Hospital Service Emergencies OR Service Emergencies, Hospital OR Service Emergency, Hospital OR Emergency Departments OR Department, Emergency OR Departments, Emergency OR Emergency Department)

### Appendix B.5. PubMed

(((((((((((((((((((((((((((risk assessment) OR (Risk Assessments)) OR (Assessment, Risk)) OR (Health Risk Assessment)) OR (Assessment, Health Risk)) OR (Health Risk Assessments)) OR (Risk Assessment, Health)) OR (Risk Analysis)) OR (Analysis, Risk)) OR (Risk Analyses)) OR (risk management)) OR (Management, Risk)) OR (Management, Risks)) OR (Risks Management)) OR (risk adjustment)) OR (Adjustment, Risk)) OR (Adjustments, Risk)) OR (Risk Adjustments)) OR (Case-Mix Adjustment)) OR (Adjustment, Case-Mix)) OR (Adjustments, Case-Mix)) OR (Case Mix Adjustment)) OR (Case-Mix Adjustments)) OR (risk factors)) OR (Factor, Risk)) OR (Risk Factor)) AND (((((early warning score) OR (Early Warning Scores)) OR (Score, Early Warning)) OR (Scores, Early Warning)) OR (Warning Scores, Early))) AND ((((((((((((((((((((((emergency service, hospital) OR (Emergency Services, Hospital)) OR (Hospital Emergency Services)) OR (Services, Hospital Emergency)) OR (Hospital Emergency Service)) OR (Service, Hospital Emergency)) OR (Emergency Hospital Service)) OR (Emergency Hospital Services)) OR (Hospital Service, Emergency)) OR (Hospital Services, Emergency)) OR (Service, Emergency Hospital)) OR (Services, Emergency Hospital)) OR (Hospital Service Emergency)) OR (Emergencies, Hospital Service)) OR (Emergency, Hospital Service)) OR (Hospital Service Emergencies)) OR (Service Emergencies, Hospital)) OR (Service Emergency, Hospital)) OR (Emergency Departments)) OR (Department, Emergency)) OR (Departments, Emergency)) OR (Emergency Department))

### Appendix B.6. Scopus

TITLE-ABS-KEY ( ( ( ( ( ( ( ( ( ( ( ( ( ( ( ( ( ( ( ( ( ( ( ( ( ( ( ( risk AND assessment ) OR ( risk AND assessments ) ) OR ( assessment, AND risk ) ) OR ( health AND risk AND assessment ) ) OR ( assessment, AND health AND risk ) ) OR ( health AND risk AND assessments ) ) OR ( risk AND assessment, AND health ) ) OR ( risk AND analysis ) ) OR ( analysis, AND risk ) ) OR ( risk AND analyses ) ) OR ( risk AND management ) ) OR ( management, AND risk ) ) OR ( management, AND risks ) ) OR ( risks AND management ) ) OR ( risk AND adjustment ) ) OR ( adjustment, AND risk ) ) OR ( adjustments, AND risk ) ) OR ( risk AND adjustments ) ) OR ( case-mix AND adjustment ) ) OR ( adjustment, AND case-mix ) ) OR ( adjustments, AND case-mix ) ) OR ( case AND mix AND adjustment ) ) OR ( case-mix AND adjustments ) ) OR ( risk AND factors ) ) OR ( factor, AND risk ) ) OR ( risk AND factor ) ) AND ( ( ( ( ( early AND warning AND score ) OR ( early AND warning AND scores ) ) OR ( score, AND early AND warning ) ) OR ( scores, AND early AND warning ) ) OR ( warning AND scores, AND early ) ) ) AND ( ( ( ( ( ( ( ( ( ( ( ( ( ( ( ( ( ( ( ( ( ( emergency AND service, AND hospital ) OR ( emergency AND services, AND hospital ) ) OR ( hospital AND emergency AND services ) ) OR ( services, AND hospital AND emergency ) ) OR ( hospital AND emergency AND service ) ) OR ( service, AND hospital AND emergency ) ) OR ( emergency AND hospital AND service ) ) OR ( emergency AND hospital AND services ) ) OR ( hospital AND service, AND emergency ) ) OR ( hospital AND services, AND emergency ) ) OR ( service, AND emergency AND hospital ) ) OR ( services, AND emergency AND hospital ) ) OR ( hospital AND service AND emergency ) ) OR ( emergencies, AND hospital AND service ) ) OR ( emergency, AND hospital AND service ) ) OR ( hospital AND service AND emergencies ) ) OR ( service AND emergencies, AND hospital ) ) OR ( service AND emergency, AND hospital ) ) OR ( emergency AND departments ) ) OR ( department, AND emergency ) ) OR ( departments, AND emergency ) ) OR ( emergency AND department ) ) )

### Appendix B.7. Cochrane Plus Collection

(risk assessment OR Risk Assessments OR Assessment, Risk OR Health Risk Assessment OR Assessment, Health Risk OR Health Risk Assessments OR Risk Assessment, Health OR Risk Analysis OR Analysis, Risk OR Risk Analyses OR risk management OR Management, Risk OR Management, Risks OR Risks Management OR risk adjustment OR Adjustment, Risk OR Adjustments, Risk OR Risk Adjustments OR Case-Mix Adjustment OR Adjustment, Case-Mix OR Adjustments, Case-Mix OR Case Mix Adjustment OR Case-Mix Adjustments OR risk factors OR Factor, Risk OR Risk Factor) AND (early warning score OR Early Warning Scores OR Score, Early Warning OR Scores, Early Warning OR Warning Scores, Early) AND (emergency service, hospital OR Emergency Services, Hospital OR Hospital Emergency Services OR Services, Hospital Emergency OR Hospital Emergency Service OR Service, Hospital Emergency OR Emergency Hospital Service OR Emergency Hospital Services OR Hospital Service, Emergency OR Hospital Services, Emergency OR Service, Emergency Hospital OR Services, Emergency Hospital OR Hospital Service Emergency OR Emergencies, Hospital Service OR Emergency, Hospital Service OR Hospital Service Emergencies OR Service Emergencies, Hospital OR Service Emergency, Hospital OR Emergency Departments OR Department, Emergency OR Departments, Emergency OR Emergency Department)

### Appendix B.8. Web of Science

((((((((((((((((((((((((((ALL = (Risk Assessment)) OR ALL = (Risk Assessments)) OR ALL = (Assessment, Risk)) OR ALL = (Health Risk Assessment)) OR ALL = (Assessment, Health Risk)) OR ALL = (Health Risk Assessments)) OR ALL = (Risk Assessment, Health)) OR ALL = (Risk Analysis)) OR ALL = (Analysis, Risk)) OR ALL = (Risk Analyses)) OR ALL = (risk management)) OR ALL = (Management, Risk)) OR ALL = (Management, Risks)) OR ALL = (Risks Management)) OR ALL = (risk adjustment)) OR ALL = (Adjustment, Risk)) OR ALL = (Adjustments, Risk)) OR ALL = (Risk Adjustments)) OR ALL = (Case-Mix Adjustment)) OR ALL = (Adjustment, Case-Mix)) OR ALL = (Adjustments, Case-Mix)) OR ALL = (Case Mix Adjustment)) OR ALL = (Case-Mix Adjustments)) OR ALL = (risk factors)) OR ALL = (Factor, Risk)) OR ALL = (Risk Factor)) AND (((((ALL = (early warning score)) OR ALL = (Early Warning Scores)) OR ALL = (Score, Early Warning)) OR ALL = (Scores, Early Warning)) OR ALL = (Warning Scores, Early)) AND ((((((((((((((((((((((ALL = (emergency service, hospital)) OR ALL = (Emergency Services, Hospital)) OR ALL = (Hospital Emergency Services)) OR ALL = (Services, Hospital Emergency)) OR ALL = (Hospital Emergency Service)) OR ALL = (Service, Hospital Emergency)) OR ALL = (Emergency Hospital Service)) OR ALL = (Emergency Hospital Services)) OR ALL = (Hospital Service, Emergency)) OR ALL = (Hospital Services, Emergency)) OR ALL = (Service, Emergency Hospital)) OR ALL = (Services, Emergency Hospital)) OR ALL = (Hospital Service Emergency)) OR ALL = (Emergencies, Hospital Service)) OR ALL = (Emergency, Hospital Service)) OR ALL = (Hospital Service Emergencies)) OR ALL = (Service Emergencies, Hospital)) OR ALL = (Service Emergency, Hospital)) OR ALL = (Emergency Departments)) OR ALL = (Department, Emergency)) OR ALL = (Departments, Emergency)) OR ALL = (Emergency Department))
